# Leukocyte ABCA1 Remains Atheroprotective in Splenectomized LDL Receptor Knockout Mice

**DOI:** 10.1371/journal.pone.0048080

**Published:** 2012-10-25

**Authors:** Bart Lammers, Ying Zhao, Amanda C. Foks, Reeni B. Hildebrand, Johan Kuiper, Theo J. C. Van Berkel, Miranda Van Eck

**Affiliations:** Division of Biopharmaceutics, Leiden/Amsterdam Center for Drug Research, Gorlaeus Laboratories, Leiden University, Leiden, The Netherlands; University of Amsterdam Academic Medical Center, The Netherlands

## Abstract

**Aim:**

ATP-binding cassette transporter A1 (ABCA1) is an important mediator of macrophage cholesterol efflux. It mediates the efflux of cellular cholesterol to lipid-poor apolipoprotein A-I. LDL receptor (LDLr) knockout (KO) mice deficient for leukocyte ABCA1 (ABCA1 KO→LDLr KO) show increased atherosclerosis and splenic lipid accumulation despite largely attenuated serum cholesterol levels. In the present study, we aimed to explore the importance of the spleen for the atheroprotective effects of leukocyte ABCA1.

**Methods:**

LDLr KO mice were transplanted with bone marrow from ABCA1 KO mice or wild-type (WT) controls. After 8 weeks recovery, mice were either splenectomized (SP-x) or underwent a sham operation, and were subsequently challenged with a Western-type diet (WTD).

**Results:**

In agreement with previous studies**,** the atherosclerotic lesion area in ABCA1 KO→LDLr KO sham animals (655±82×10^3^ µm^2^) was 1.4-fold (p = 0.03) larger compared to sham WT→LDLr KO mice (459±33×10^3^ µm^2^) after 8 weeks WTD feeding, despite 1.7-fold (p<0.001) lower serum cholesterol levels. Interestingly, deletion of ABCA1 in leukocytes led to 1.6-fold higher neutrophil content in the spleen in absence of differences in circulating neutrophils. Levels of KC, an important chemoattractant for neutrophils, in serum, however, were increased 2.9-fold (p = 0.07) in ABCA1 KO→LDLr KO mice. SP-x induced blood neutrophilia as compared to WT→LDLr KO mice (1.9-fold; p<0.05), but did not evoke differences in serum cholesterol and anti-oxLDL antibody levels. Atherosclerotic lesion development, however, was 1.3-fold induced both in the presence and absence of leukocyte ABCA1 (WT: 614±106×10^3^ µm^2^, ABCA1 KO: 786±44×10^3^ µm^2^). Two-way ANOVA revealed independent effects on atherosclerosis for both leukocyte ABCA1 deficiency and SP-x (p<0.05).

**Conclusions:**

The observed splenic alterations induced by leukocyte ABCA1 deficiency do not play a significant role in the anti-atherogenic effects of leukocyte ABCA1 on lesion development.

## Introduction

Reverse cholesterol transport (RCT) is an important mechanism by which HDL and its major apolipoprotein A-I (apoA-I) protect against atherosclerosis. [1] In this process, the cellular cholesterol efflux machinery is essential to maintain cellular lipid homeostasis in macrophages and to prevent pathological foam cell formation, a hallmark of atherosclerosis. A key regulator of macrophage cholesterol efflux is ATP-binding cassette (ABC) transporter ABCA1, which facilitates cholesterol efflux to lipid-poor apolipoproteins like apoA-I, [2] thereby initiating the generation of HDL. [3], [4] Deficiency of leukocyte ABCA1 on the LDLr KO background (ABCA1 KO→LDLr KO) led to increased atherosclerosis, despite largely attenuated cholesterol levels. [5] Interestingly, these mice also showed elevated leukocyte counts in the circulation, [6] and accumulation of macrophages in the peritoneal cavity, liver, and spleen. [5] This indicates that leukocyte ABCA1, in addition to its role in cholesterol efflux, exerts regulatory functions in the recruitment of inflammatory cells to the periphery.

The spleen is the largest lymphoid organ in the body with important immunological functions. It produces antibodies, facilitates phagocytosis and is capable of eliminating foreign antigens. [7], [8] However, it also serves as a blood filter by removing old and abnormal red blood cells, [9] and functions as an important monocyte reservoir. [10] Since atherosclerosis is believed to result from a combination of dyslipidemia and vascular inflammation, [11] the role of the spleen with respect to atherosclerosis and serum lipid levels has been thoroughly investigated.[12]–[16] It has been previously reported that total cholesterol (TC) levels increase after splenectomy. [12], [13] However, Western-type diet fed, splenectomized apoE KO mice display increased atherosclerosis as compared to sham-operated controls, without changes in TC levels. [15], [17].

To investigate the possible interplay between the spleen and leukocyte ABCA1 with respect to the development of atherosclerosis, we transplanted bone marrow from ABCA1 deficient mice into LDLr deficient recipient mice, which were subsequently either splenectomized or underwent a sham operation. Our results evidently show that leukocyte ABCA1 deficiency resulted in decreased TC levels, increased inflammation, and lipid and neutrophil accumulation in the spleen. However, the observed splenic alterations induced by leukocyte ABCA1 deficiency did not alter anti-oxLDL antibody levels, nor played a significant role in atherosclerotic lesion development as evidenced by splenectomy.

## Methods

### Animals, Bone Marrow Transplantation, and Splenectomy

Animal experiments were approved by the Ethics Committee for Animal Experiments of Leiden University (permit number 09171) and performed at the Gorlaeus Laboratories of the Leiden/Amsterdam Center for Drug Research in accordance with the National Laws and the Directive 2010/63/EU of the European Parliament.

C57BL/6J mice and ABCA1 KO [18] mice (more than 7 times backcrossed onto a C57BL/6J background) were used as donors for the bone marrow transplantation. These donor mice were anaesthetized by subcutaneous injection with a mix of 70 mg/kg body weight xylazine, 1.8 mg/kg bodyweight atropine and 350 mg/kg body weight ketamine. Animals were subsequently sacrificed by cervical dislocation. Homozygous C57BL/6J LDL receptor knockout (LDLr KO) mice were obtained from The Jackson Laboratory as mating pairs and bred at the Gorlaeus Laboratories, Leiden, The Netherlands. Bone marrow transplantations into LDLr KO mice were performed as described. [19] Briefly, irradiated recipients (≥11 per group) received 5×10^6^ bone marrow cells by intravenous injection into the tail vein. After 8 weeks, mice were either splenectomized (SP-x) or underwent a sham operation. Mice were anesthetized with isoflurane inhalation. After anesthesia, mice were surgically prepared by first shaving the incision site, followed by preparing the incision site with alcohol. A small incision was made in the left subcostal abdominal wall, through which the spleen was exteriorized. Splenectomy was performed by placing ligatures around the splenic vasculature and subsequently removing the spleen. The incision was closed in two layers using surgical sutures. Mice were monitored for recovery from anesthesia and kept at 37°C until wakeup. Control mice underwent a sham operation and were maintained in the same conditions.

After a recovery period of 2 weeks, the animals were challenged with a Western-type diet (WTD; 0.25% cholesterol and 15% cocoa butter; Special Diet Services, Witham, Essex, UK) for 8 weeks to induce atherosclerotic lesion development. At 18 weeks after transplantation, mice were anaesthetized by subcutaneous injection with a mix of 70 mg/kg body weight xylazine, 1.8 mg/kg bodyweight atropine and 350 mg/kg body weight ketamine. Animals were subsequently sacrificed by cervical dislocation.

### Histological Analysis of the Aortic Root and Spleen

To analyze the development of atherosclerosis at the aortic root, transplanted LDLr KO mice were euthanized 18 weeks after bone marrow transplantation. The arterial tree was perfused in situ with PBS (100 mm Hg) for 10 min via a cannula in the left ventricular apex. The heart plus aortic root and the aortic arch were excised and stored in 3.7% neutral-buffered formalin (Formal-fixx; Shandon Scientific Ltd, Runcorn, UK). Serial sections (10 µm) of the aortic root were cut using a Leica CM3050S cryostat. The atherosclerotic lesion areas in oil red-O stained cryostat sections of the aortic root were quantified using the Leica image analysis system, consisting of a Leica DMRE microscope coupled to a video camera and Leica Qwin Imaging software (Leica Ltd, Cambridge, UK). Mean lesion area (in µm^2^) was calculated from 10 consecutive oil red-O stained sections of the aortic root, starting at the appearance of the tricuspid valves. Collagen content of the lesions was determined after Masson Trichrome staining (Sigma diagnostics, St Louis, MO). Furthermore, acellular areas were quantified as the area lacking nuclei in Masson Trichrome/hematoxylin-stained sections. Sections were stained immunohistochemically for the presence of neutrophils and macrophages using a rat Ly6G antibody (monoclonal rat IgG2b, dilution 1∶100; eBioscience, San Diego, CA), and a MoMa-2 antibody (dilution 1∶50; Serotec Ltd, Oxford, UK), respectively. A goat anti-rat antibody coupled to horse radish peroxidase (HRP) (1∶100) (Dako, Glostrup, Denmark) was used as a secondary antibody and Nova red substrate (Vector Laboratories, Burlingame, California) was used for visualization of HRP. In addition, 7 µm cryosections of formalin-fixed spleen from the transplanted sham-operated LDLr KO mice were prepared and stained for neutrophils using a rat Ly6G antibody (monoclonal rat IgG2b, dilution 1∶100; eBioscience, San Diego, CA), and counterstained with hematoxylin.

### Lipid Analysis

At 8 weeks after bone marrow transplantation, 100 µL of blood was drawn from each individual mouse by tail bleeding after an overnight fasting period. Upon sacrifice, 18 weeks after bone marrow transplantation, blood was collected by retro-orbital venous plexus puncture after an overnight fasting period. Total cholesterol and triglyceride analyses were performed as described. [19].

### Serum Antibody Detection

Murine monocyte chemoattractant protein-1 (MCP-1; BD Biosciences, Erembodegem, Belgium) and keratinocyte chemoattractant (KC; Biosource) serum levels were assayed using an ELISA kit according to the manufacturer’s protocol. IgM and IgG2a levels against oxLDL were detected in serum using antibodies recognizing mouse IgM/IgG2a and HRP-labeled goat anti-rat Ig (BD Pharmingen). OxLDL (5 µg/mL) was dissolved in NaHCO_3_/Na_2_CO_3_ buffer (pH 9.6) and was coated overnight onto a flat-bottom 96-well high binding plate (Corning, New York, USA). Absorbance was detected at 450 nm.

### Flow Cytometry

Upon sacrifice, blood was collected from the transplanted animals. Erythrocytes were lysed using erythrocyte lysis buffer (0.15M NH_4_Cl, 10 mM NaHCO_3_, 0.1 mM EDTA, pH 7.3). For the detection of CD11b^+^GR-1^+^ neutrophils, the blood cells were stained for the surface markers CD11b and GR-1 (0.25 µg Ab/200,000 cells). Antibodies were purchased from eBioscience, Vienna, Austria). Fluorescent activated cell sorting (FACS) analyses were performed on a FACS Canto II (BD Biosciences, Mountain View, CA). Data were analyzed using FACSDiva software (BD Biosciences).

### Statistical Analysis

Statistically significant differences among the means of the different populations were tested using analysis of variance (ANOVA) and when specifically indicated the unpaired Student’s t-test (GraphPad InStat and Prism software). The Student-Newman-Keuls multiple comparison test was performed after ANOVA. Two-way ANOVA was used to check possible interactions. The probability level (alpha) for statistical significance was set at 0.05. Results are expressed as an average ± SEM.

## Results

### Increased Splenic and Systemic Inflammation in ABCA1 KO**→**LDLr KO Mice

**Figure 1:**
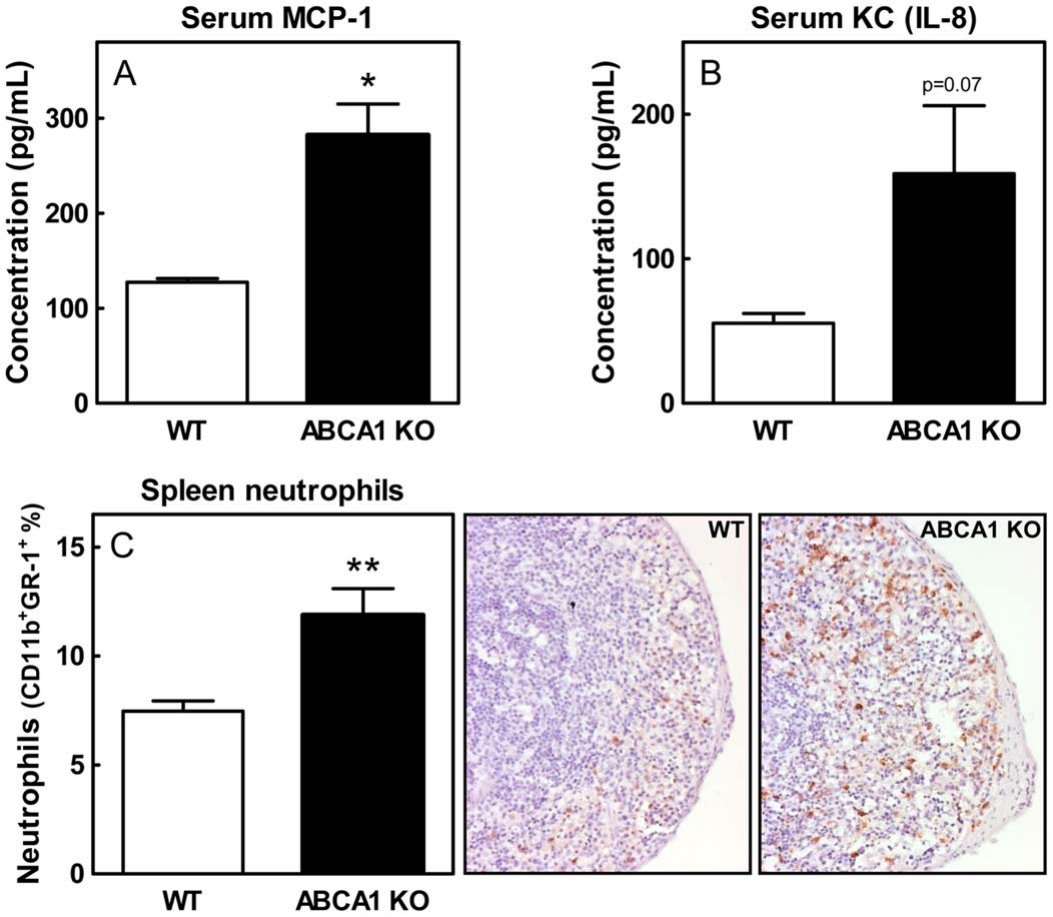
Highly induced concentrations of the pro-inflammatory cytokines MCP-1 and KC (murine IL-8) in serum and increased splenic neutrophil content in ABCA1 KO→LDLr KO mice. Serum was obtained from WTD fed WT→LDLr KO and ABCA1 KO→LDLr KO mice. Serum MCP-1 and KC levels were determined by ELISA (A, B). n≥4 mice ± SEM per group. After red blood cell lysis, splenic neutrophil content (CD11b^+^GR-1^+^ cells) was analyzed by FACS (C, left panel). Values represent the percentage of total spleen cells. n≥6 mice ± SEM per group. Representative spleen sections were stained with an anti-Ly6G antibody (original magnification 100x; C, right panels). *p<0.05, **p<0.01.

Deficiency of leukocyte ABCA1 has been shown to induce the number of leukocytes in the circulation. In addition, the spleens of these mice exhibited increased macrophage accumulation. [6] In the current study, we observed induced concentrations of the proinflammatory cytokines MCP-1 (2.2-fold; p<0.05), and KC (murine IL-8; 2.9-fold; p = 0.07) in serum of ABCA1 KO→LDLr KO mice (Fig. 1). Interestingly, IL-8 is one of the most potent chemoattractants for neutrophils. [20], [21] In agreement, spleen sections stained for Ly6G revealed increased neutrophil presence in spleens from ABCA1 KO→LDLr KO mice. This observation was confirmed by FACS analysis on the spleen, which showed that the splenic neutrophil content was increased 1.6-fold (p<0.01) in ABCA1 KO→LDLr KO mice compared to WT→LDLr KO mice (Fig. 1). In order to establish the importance of the spleen for the atheroprotective effects of leukocyte ABCA1, we subjected WT→LDLr KO and ABCA1 KO→LDLr KO mice to splenectomy.

### No Effects of Splenectomy on Serum Total Cholesterol, Triglyceride and Anti-oxLDL Levels

Cholesterol levels were measured at 8 weeks (chow diet) and at 18 weeks (WTD, containing 0.25% cholesterol and 15% cocoa butter) after bone marrow transplantation (BMT; Table 1). On chow diet, total cholesterol (TC) levels were not different between the groups. As expected, after feeding WTD, serum TC levels of WT→LDLr KO mice increased ≈4.5-fold. However, in agreement with previous studies, [6], [22], [23] deletion of ABCA1 in bone marrow cells resulted in an attenuated increase in plasma TC levels upon feeding the atherogenic diet (≈2.9-fold as compared to both SP-x (p<0.05) and sham operated controls (p<0.01)). No differences were observed between splenectomized animals and sham operated controls. In addition, during WTD feeding triglyceride (TG) levels were unaffected in all experimental groups (Table 1). Furthermore, splenectomized mice did not show altered antibody titers of anti-oxLDL IgM and IgG2a on WTD (Table 1). ABCA1 KO→LDLr KO mice exhibited slightly decreased anti-oxLDL antibody titers as compared to WT→LDLr KO mice. However, these values did not reach statistical significance. These data indicate that splenectomy did not induce significant changes in TC, TG, and anti-oxLDL antibody response.

**Table 1:** Serum TC, TG and anti (α)-oxLDL antibody levels were measured at 10 and/or 18 weeks after BMT.

DonorBoneMarrow	Time(wks)	Diet	BodyWeight(g)	Total Cholesterol(mg/dL)	Trigly-cerides(mg/dL)	α-oxLDLIgM(OD)	α-oxLDLIgG2a(OD)
WT→LDLr KO Sham	10	Chow	29±0.6	354±26	n.d.	n.d.	n.d.
	18	WTD	27±0.5	1137±71	68±9	1.10±0.38	0.25±0.01
ABCA1 KO→ LDLr KO sham	10	Chow	28±0.5	323±5	n.d.	n.d.	n.d.
	18	WTD	29±1.3	676±30**	62±11	0.85±0.47	0.17±0.02
WT →LDLr KO SP-x	10	Chow	28±0.7	299±17	n.d.	n.d.	n.d.
	18	WTD	27±0.8	1144±177	74±6	1.26±0.15	0.27±0.05
ABCA1 KO →LDLr KO SP-x	10	Chow	28±1.0	247±19**	n.d.	n.d.	n.d.
	18	WTD	28±1.4	713±54*	100±26	0.88±0.07	0.21±0.08

Data represent mean ± SEM of ≥10 mice. *p<0.05, **p<0.01, compared to respective WT. n.d. indicates not determined.

### Increased Neutrophil Levels in Splenectomized ABCA1 KO→LDLr KO Mice

**Figure 2:**
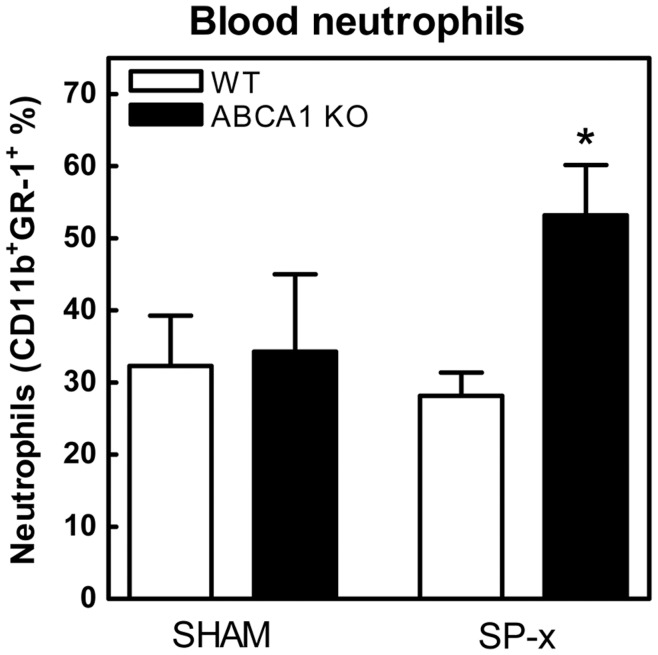
Increased levels of circulating neutrophils in splenectomized ABCA1 KO→LDLr KO mice. At 18 weeks after BMT, blood was drawn from WT→LDLr KO and ABCA1 KO→LDLr KO mice. After red blood cell lysis, neutrophil content (CD11b^+^GR-1^+^ cells) was analyzed by FACS. Values are the percentage of total white blood cell counts. n≥3 mice ± SEM per group. *p<0.05 compared to splenectomized controls.

Upon sacrifice after 8 weeks of WTD feeding, FACS analysis was performed to measure MCP-1 and KC levels, as well as neutrophil content in the blood. Splenectomy did not result in differences in MCP-1 and KC levels as compared to controls. Although ABCA1 KO→LDLr KO mice exhibited increased splenic neutrophil content, no differences in neutrophil levels were observed in blood between WT→LDLr KO and ABCA1 KO→LDLr KO sham operated animals. Upon splenectomy, however, ABCA1 KO→LDLr KO mice displayed a 1.9-fold increase in neutrophil levels in blood compared to splenectomized WT→LDLr KO mice (p<0.05; Fig. 2).

### Increased Atherosclerosis in Splenectomized Transplanted LDLr KO Mice

**Figure 3:**
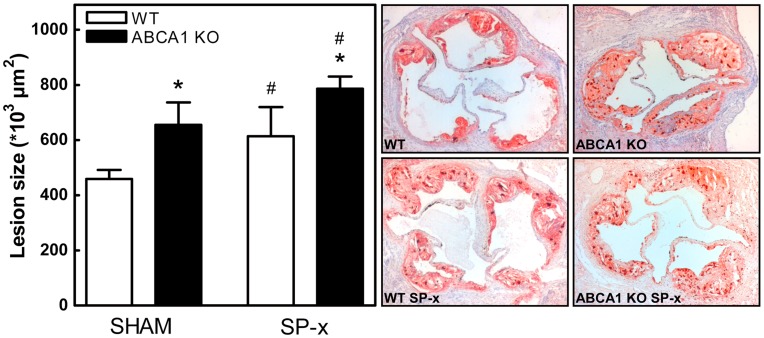
Independent effects on atherosclerosis for both leukocyte ABCA1 deficiency and splenectomy. Quantification of atherosclerotic lesion sizes of WT→LDLr KO and ABCA1 KO→LDLr KO mice after 8 weeks of WTD feeding (left panel). Representative oil red-O stained cross-sections (original magnification 50x; right panels). Values represent the means of the average lesion size in 10 consecutive aortic root sections per mouse. n≥8 mice ± SEM per group. *p<0.05 compared to respective WT controls; #p<0.05 compared to sham operated controls.

**Figure 4:**
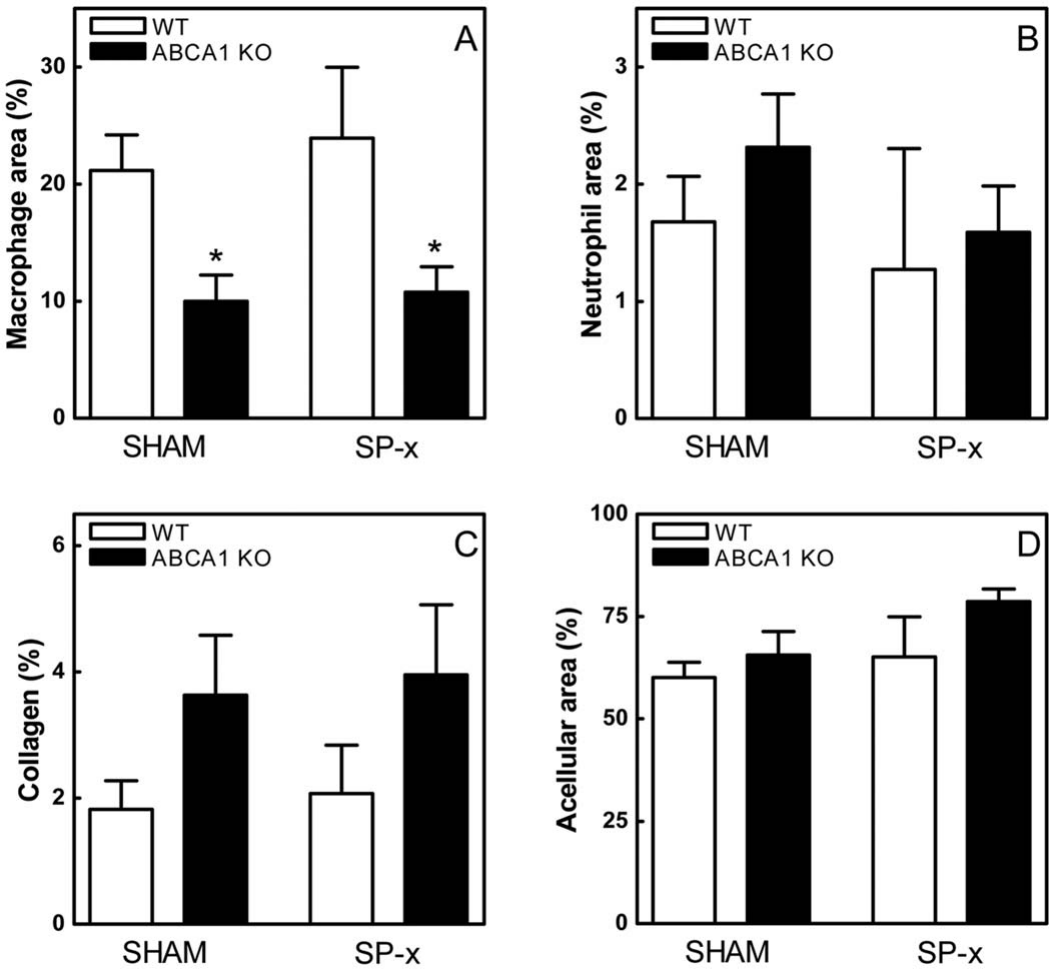
Decreased macrophage content per lesion area in atherosclerotic lesions from ABCA1 KO→LDLr KO mice. Representative slides were stained with MoMa-2 antibody, anti-Ly6G antibody, and Masson Trichrome for the detection and quantification of macrophages (A), neutrophils (B), and collagen (C) content, respectively, after 8 weeks of WTD feeding. Lesional acellular areas were quantified as the area lacking nuclei in Masson Trichrome/hematoxylin-stained sections (D). Values represent the means of 5 consecutive aortic root sections of individual mice and are expressed as the percentage stained area per lesion area. n≥4 mice ± SEM per group. *p<0.05 compared to respective WT controls.

Atherosclerotic lesion size and composition in the aortic root of the transplanted animals were analyzed after feeding the mice a WTD for 8 weeks. As expected, quantification of the lesion sizes in oil red-O stained sections of the aortic root of sham-operated animals showed that ABCA1 KO→LDLr KO mice exhibited a 1.4-fold increase (t-test; p = 0.03) in the mean atherosclerotic lesion size compared to lesions from WT→LDLr KO mice (655±82×10^3^ µm^2^ and 459±33×10^3^ µm^2^, respectively; Fig. 3). Splenectomy induced a 1.2- and 1.3-fold increase in atherosclerotic lesion formation in ABCA1 KO→LDLr KO mice (786±44×10^3^ µm^2^) and WT→LDLr KO mice (614±106×10^3^ µm^2^), respectively (Fig. 3). In ABCA1 KO→LDLr KO mice the percentage of macrophages per lesion area was attenuated (p<0.05; Fig. 4), indicative of more advanced lesions. The acellular regions as well as the neutrophil and collagen content per lesion area were not changed significantly (Fig. 4), although the absolute size of the acellular regions (WT→LDLr KO SHAM: 296±32×10^3^ µm^2^, ABCA1 KO→LDLr KO SHAM: 481±80×10^3^ µm^2^, WT→LDLr KO SP-x: 399±89×10^3^ µm^2^, ABCA1 KO→LDLr KO SP-x: 597±11×10^3^ µm^2^; p = 0.01), neutrophil and collagen positive area (data not shown) was increased, in agreement with the overall increased lesion size. Two-way ANOVA was used to verify the independent effects of leukocyte ABCA1 deficiency and splenectomy on atherosclerotic lesion development. This test showed significant, independent contributions of both leukocyte ABCA1 deficiency (p = 0.012) and splenectomy (p = 0.048) to the observed increases in atherosclerotic lesion development, demonstrating the particular importance of both leukocyte ABCA1 and the spleen with respect to atherosclerosis.

## Discussion

The importance of ABCA1 in cellular cholesterol transport became clear when mutations in the ABCA1 gene were discovered to cause Tangier disease, an HDL deficiency disorder. [24] Additional research revealed a critical role for ABCA1 in leukocytes, as it was shown to facilitate macrophage cholesterol efflux. [25] As a consequence, deficiency of ABCA1 in leukocytes resulted in increased atherosclerotic lesion development. [6], [26] However, more recent findings also suggested an anti-inflammatory role for leukocyte ABCA1 in atherosclerosis.[27]–[29] In agreement, the current study showed highly induced concentrations of MCP-1 and KC (murine IL-8) in the serum of ABCA1 KO→LDLr KO mice fed a WTD. MCP-1 is an important chemoattractant for mononuclear cells. Mice deficient for MCP-1 or its receptor chemokine receptor 2 (CCR-2) develop fewer and smaller atherosclerotic lesions than control mice, as a consequence of the reduced ability to recruit monocytes to sites in the arterial wall prone to atherosclerotic lesion development.[30]–[32] KC (murine IL-8) triggers monocyte arrest on early atherosclerotic endothelium, [33] and plays a central role in macrophage accumulation in established fatty streak lesions. [34] The increased KC and MCP-1 levels in mice lacking ABCA1 seem to be an apparent contradiction with the observed decrease in the amount of macrophages per lesion area, but this is likely the consequence of the fact that mice lacking leukocyte ABCA1 develop more advanced lesions. Interestingly, IL-8 is also one of the most potent chemoattractants for neutrophils. [20], [21] Neutrophils are short-lived phagocytic cells that serve as essential early cellular effectors of innate immunity and constitute the “first line of defense”. Accordingly, ABCA1 KO→LDLr KO mice exhibited increased neutrophil presence in the spleen. Upon splenectomy, ABCA1 KO→LDLr KO mice developed blood neutrophilia as compared to WT→LDLr KO mice. Surprisingly, leukocyte ABCA1 deficiency or splenectomy alone did not alter blood neutrophil concentrations. In response to inflammatory processes, neutrophils are rapidly mobilized from the bone marrow, creating a blood neutrophilia. [35] Following their accumulation at sites of inflammation, neutrophils become apoptotic and are efficiently cleared, primarily by the liver and the spleen, [36] in order to prevent excessive tissue damage. The observed blood neutrophilia in splenectomized ABCA1 KO→LDLr KO mice might be the combined result of a chronic advanced inflammatory status because of the lack of macrophage ABCA1, and absence of splenic neutrophil clearance.

Of note, despite the increased presence of neutrophils in the circulation of splenectomized ABCA1 KO→LDLr KO mice, no differences were observed in the amount of neutrophils per lesion area present in atherosclerotic plaques of these mice. The half-life of the neutrophils in the plaque might be too short to allow detection of increased neutrophil presence. [37] On the other hand, differential results on the relation between lesional and circulating neutrophils have recently been published, [38], [39] indicating that higher levels of circulating neutrophils do not always result in increased numbers of lesional neutrophils.

The spleen is also associated with systemic immune responses in which it is the principal organ responding to antigens such as oxLDL. [17] OxLDL deposits in the arterial wall are believed to be involved in the initiation of atherosclerosis by damaging the vascular endothelium and engulfment by macrophages. [40] Accordingly, anti-oxLDL antibody serum titers have been suggested to play an anti-atherogenic role. [17] Anti-oxLDL antibody production, however, might also be increased as a result of enhanced inflammation. No differences in anti-oxLDL antibody serum titers were observed upon splenectomy. Deficiency of leukocyte ABCA1 resulted in a moderate decrease in anti-oxLDL antibody serum titers. This might be the direct result of the attenuated serum TC levels in ABCA1 KO→LDLr KO mice, since serum TC levels have been positively correlated to serum titers of anti-oxLDL antibodies. [41].

The importance of the spleen in lipid metabolism has been investigated previously.[12]–[14] However, differential results were obtained regarding serum TC and TG concentrations after splenectomy. Splenectomized apoE KO mice displayed increased atherosclerotic lesion development as compared to their sham operated littermates, in absence of changes in serum TC levels. [15] As expected, the current study revealed attenuated TC levels in ABCA1 KO→LDLr KO mice. However, no differences in TC levels were observed as a result of splenectomy.

Despite the fact that splenectomy did not lead to differences in either serum TC and TG levels, or anti-oxLDL antibody titers, splenectomized mice did show enhanced atherosclerotic lesion development. As expected, ABCA1 KO→LDLr KO mice also revealed increased atherosclerotic lesion formation. Moreover, splenectomized ABCA1 KO→LDLr KO mice exhibited an additional increment in lesion development. These results suggest that the observed splenic alterations induced by leukocyte ABCA1 deficiency do not play a significant role in the anti-atherogenic effects of leukocyte ABCA1. Leukocyte ABCA1 deficiency, as well as splenectomy independently induce atherosclerotic lesion development, demonstrating the particular importance of both leukocyte ABCA1 and the spleen with respect to atherosclerosis.
